# Estimation of Cardiac Short Axis Slice Levels with a Cascaded Deep Convolutional and Recurrent Neural Network Model

**DOI:** 10.3390/tomography8060229

**Published:** 2022-11-14

**Authors:** Namgyu Ho, Yoon-Chul Kim

**Affiliations:** 1 Kim Jaechul Graduate School of Artificial Intelligence, KAIST, Seoul 02455, Republic of Korea; 2 Department of Computer Science and Engineering, Sogang University, Seoul 04107, Republic of Korea; 3 Division of Digital Healthcare, College of Software and Digital Healthcare Convergence, Yonsei University, Wonju 26493, Republic of Korea

**Keywords:** deep learning, cardiac imaging, convolutional neural network, recurrent neural network

## Abstract

Automatic identification of short axis slice levels in cardiac magnetic resonance imaging (MRI) is important in efficient and precise diagnosis of cardiac disease based on the geometry of the left ventricle. We developed a combined model of convolutional neural network (CNN) and recurrent neural network (RNN) that takes a series of short axis slices as input and predicts a series of slice levels as output. Each slice image was labeled as one of the following five classes: out-of-apical, apical, mid, basal, and out-of-basal levels. A variety of multi-class classification models were evaluated. When compared with the CNN-alone models, the cascaded CNN-RNN models resulted in higher mean F1-score and accuracy. In our implementation and testing of four different baseline networks with different combinations of RNN modules, MobileNet as the feature extractor cascaded with a two-layer long short-term memory (LSTM) network produced the highest scores in four of the seven evaluation metrics, i.e., five F1-scores, area under the curve (AUC), and accuracy. Our study indicates that the cascaded CNN-RNN models are superior to the CNN-alone models for the classification of short axis slice levels in cardiac cine MR images.

## 1. Introduction

Cardiac imaging has been developed in a sophisticated way to investigate morphological and functional characteristics of the heart in vivo [1]. Echocardiography is safe and portable, but it has limitations in its dependence on operators’ skills and low image quality, especially in deep structures. Computed tomography (CT) is primarily used in imaging the coronary arteries but induces radiation exposure in patients. Magnetic resonance imaging (MRI) may pose a risk in patients with cardiac implantable devices [2], but it allows for imaging of an arbitrary scan plane orientation and provides excellent contrast between the myocardium and blood. Cardiac short axis slice imaging is performed to evaluate the heart wall motion in cardiac cine MRI, which acquires the entire left ventricle (LV) in a slice-by-slice manner with high temporal resolution [3]. Knowledge of the short axis slice level is necessary for either reporting findings of a cardiac MR exam or visualizing the regional analysis results on the 17 myocardial segment model [4].

Automatic classification of cardiac slice orientation has been investigated in the literature. A fine-tuned convolutional neural network (CNN) was used to identify a slice orientation out of the five orientations, which are two chamber, three chamber, four chamber, LV outflow tract, and short axis [5]. A deep CNN was used to identify a slice out of the apical slice or a slice out of the basal slice [6]. A fine-tuned deep CNN model was used to identify a short axis slice level out of the three classes, which are out-of-apical, in, and out-of-basal levels [7]. These approaches did not investigate the feasibility of differentiating apical, mid, and basal slice levels, and they were developed to predict an output class given a single image as input. In cardiac cine MRI, the entire LV is acquired by imaging a stack of short axis slices from the apex to the base, and the slices from acquired data are ordered in a sequence of the apical, mid, and basal levels. Hence, it would be desirable to consider a model that takes the adjacent slice information into account. A hybrid of a CNN and a recurrent neural network (RNN) may be well suited to the classification of cardiac short axis slice levels from cardiac cine MRI.

**Figure 1:**
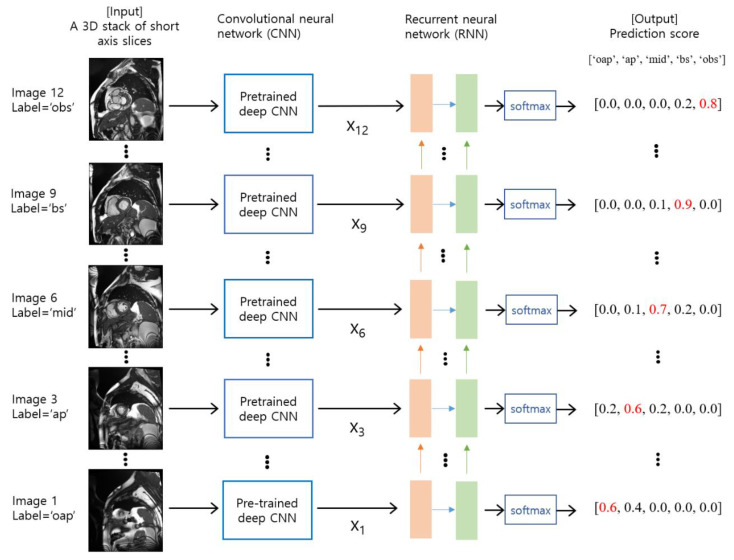
An overview of the proposed method. The input consists of a series of cardiac short axis slice images, which range from the out-of-apical slice level to the out-of-basal slice level. The slice levels were labeled as ‘oap’ for the out-of-apical slice, ‘ap’ for the apical slice, ‘mid’ for the mid-level slice, ‘bs’ for the basal slice, and ‘obs’ for the out-of-basal slice. For each slice image, a pre-trained deep CNN model is used as a feature extractor. The features are denoted by x_i_ for i = 1, 2, …, N, where N is the number of slices. The RNN model takes a series of features (i.e., x_1_, x_2_, …, x_N_) as input and produces probability scores for each slice level via softmax. The red numbers indicate the maximum values in the output prediction scores.

In this study, we investigate a many-to-many RNN model for the cardiac slice classification problem, where baseline CNNs are used for feature extractors and an RNN model is used for modeling the sequence of slice levels, as described by Figure 1. It is noted that the hybrid models of CNN and RNN have been developed for image classification in medical image analysis [8,9,10,11,12]. We evaluate the performance of different types of cascaded CNN-RNN models in terms of accuracy and F1-scores for the prediction of short axis slice level. We also compare the performance of a cascaded CNN-RNN model against a CNN-alone model that takes a single image as input and predicts an output class.

## 2. Materials and Methods

This section describes procedures of data curation, data labeling, deep learning model training and validation, and evaluation on unseen test data. Code related to this study is available at https://github.com/itsnamgyu/mri-classification (accessed on 6 November 2022).

### 2.1. Dataset

In the present study, we used publicly available data from the Kaggle’s 2015 Data Science Bowl challenge (https://www.kaggle.com/competitions/second-annual-data-science-bowl/overview) (accessed on 6 November 2022). The Kaggle’s 2015 Data Science Bowl challenge data have short axis cardiac cine MRI Digital Imaging and Communications in Medicine (DICOM) data of a total of 1140 subjects’, which consist of 500 subjects’ data for training, 200 subjects’ data for validation, and 440 subjects’ data for testing. To ensure a reliable study, we filtered out unreliable data. Out of the 1140 subjects’ data, we excluded 166 subjects’ data, which had one of the following issues: (1) multiple images existed with the same identifiers, (2) the number of slices from different phases was inconsistent, or (3) the slice indices were non-contiguous within a given phase. For instance, the slice index 3 was missing in a series of 10 slices. We converted the DICOM data to png files after changing the image intensity to the [0 255] range. After the exclusion of 166 subjects, we randomly assigned the remaining 974 subjects’ data into either training, validation, or testing data, as shown in Table 1. This assignment was performed subject-wise. The cardiac cine MRI dataset has 20 to 30 dynamic frames per slice and 8 to 25 slices per subject. Out of 20 to 30 dynamic frames, we considered two cardiac phases: one diastolic phase and one systolic phase [13]. The total sample sizes for CNN and CNN-RNN models are shown in Table 2.

### 2.2. Data Labeling

To label the images, we developed a custom user interface which was implemented using the Matplotlib [14] library for image slice labeling in a diastolic and a systolic frame for all subjects. The user interacted with the user interface to classify each short axis slice into one of the following five categories: (1) out-of-apical, (2) apical, (3) mid, (4) basal, and (5) out-of-basal slice levels. Out-of-apical was defined as a slice that shows no appearance of the LV blood pool. Out-of-basal was defined as a slice above the most basal slice that is characterized by a small crescent of basal lateral myocardium and no discernable LV blood pool [15]. Apical, mid, and basal levels were defined as follows. The apical-level slice corresponds to an image which is adjacent to the apex and has no papillary muscle. The mid-level slice corresponds to an image with papillary muscle. The basal-level slice corresponds to an image which is adjacent to the base and has no papillary muscle. As a recommended consensus, the LV volume is divided into basal, mid, and apical levels with identical thickness [16], but our study adopted the slice-level identification based on the presence or absence of the papillary muscle. The labeling results were saved upon closing the interface. They were saved in an internal metadata file, which was reloaded when the user resumed the manual labeling task. The number of images for each class is listed in Table 3.

### 2.3. Deep Learning Model Training and Validation

Table 4 lists the networks considered in our study, including their model capacities, the ImageNet top-1 accuracy scores, the number of penultimate features, and batch sizes for the CNN and CNN-RNN models. We applied transfer learning in the fixed-feature extractor setting. We used the penultimate features from the convolutional base of a CNN model as input to a custom deep neural network (DNN) classifier. The custom DNN classifier consisted of a fully connected layer with 256 units as output, a ReLu activation, a dropout layer with a dropout rate of 0.5 [17], a fully connected layer with five units as output, and softmax activation. We simply appended the custom DNN classifier to the existing base network and froze the base convolutional layer weights during training. The final softmax layer had five output nodes that corresponded to the five classes in our classification task: out-of-apical, apical, mid, basal, and out-of-basal. The Keras application library (https://www.tensorflow.org/api_docs/python/tf/keras/applications) (accessed on 6 November 2022) [18]. on the Tensorflow version 2.8.0 was used to compare the performance of a variety of pre-trained deep CNN models, in which the weights were trained on the ImageNet dataset [19].

**Figure 2:**
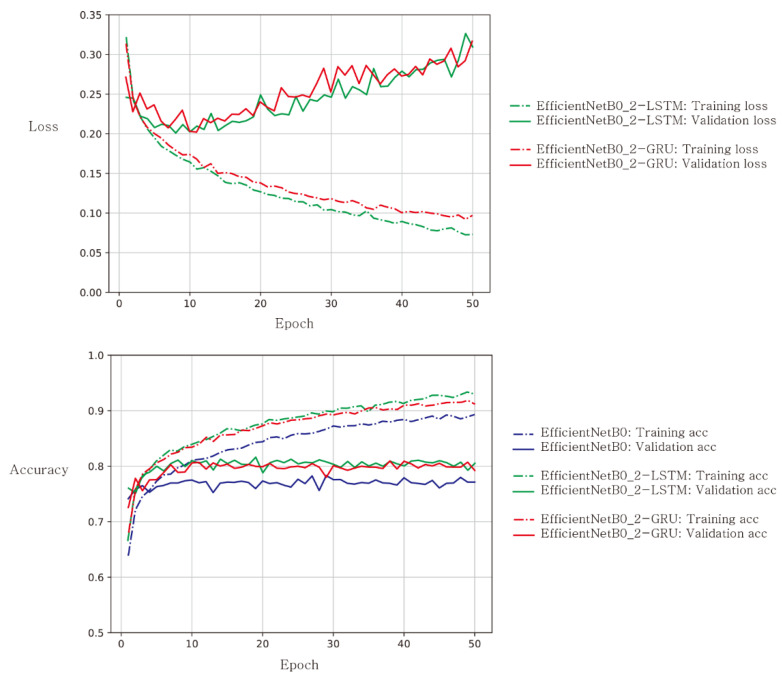
Learning curves for loss (**top**) and accuracy (**bottom**) when EfficientNetB0 was used as a baseline CNN architecture. Solid lines represent validation results, while dashed lines represent training results. Loss curves for EfficientNetB0 were not plotted because their loss value ranges were well above those for EfficientNetB0_2-LSTM and EfficientNetB0_2-GRU.

Training and validation were performed on a single GPU (NVIDIA GeForce GTX 1080 with 8 GB memory). To train the network, we used the mini-batch gradient descent optimization with a batch size of 32 and the Adam optimizer [20]. Four deep CNN base networks were considered: EfficientNetB0 [21], MobileNet [22], NASNetMobile [23], and ResNet50V2 [24]. We tested four different types of RNNs: two-layer long short-term memory (LSTM) [25], bidirectional LSTM [26], two-layer gated recurrent unit (GRU) [27], and bidirectional GRU. Each hidden layer had 128 units in each hidden cell and consisted of 25 hidden cells, where 25 is the number of time steps. We set the number of time steps to 25 because the maximum number of short axis slices was 25 in our dataset. Categorical cross-entropy was adopted as a loss function. We trained each model for 50 epochs, as shown in Figure 2, and selected an appropriate epoch number based on manual inspection of the validation loss curve. To avoid overfitting, we chose the appropriate epoch value from the validation loss curve where the validation loss function was near the minimum.

### 2.4. Evaluation

A total of 20 models were evaluated: 4 models obtained by training a custom DNN classifier on top of base CNNs used for feature extraction and 16 models obtained by the cascade of CNN and RNN. Performance was evaluated against the test dataset of 184 patients that was held out during model development. We used the scikit-learn library [28] to calculate the following: F1-score, area under the curve (AUC), accuracy, and confusion matrix. For the calculation of F1-score, we considered the one-vs.-rest classification task for each class. For example, by regrouping the classes into ‘mid’ and ‘non-mid’, where the ‘non-mid’ group consists of ‘oap’, ‘ap’, ‘bs’, and ‘obs’, we obtained binary classification for the ‘mid’ group. We applied this approach to all five classes and evaluated precision, recall, F1-score, and AUC. The precision was the number of true positive cases divided by the number of all positive predicted cases. The recall was the number of true positive cases divided by the number of all positive cases. The F1-score was calculated as the harmonic mean of the precision and recall. The accuracy was the number of correctly predicted cases divided by the number of all cases. In addition, we counted the sum of the elements out of the tridiagonal entries in the confusion matrix and termed it SOTD. We used SOTD for the evaluation since it reflects obvious misclassification errors made by a deep learning model.

## 3. Results

As shown in Table 3, in the training dataset, the ‘ops’, ‘ap’, ‘mid’, ‘bs’, and ‘obs’ classes were 15.5, 23.1, 23.2, 17.7, and 20.5%, respectively, A similar class distribution was observed in the validation dataset. The class imbalance was not severe in this study.

Figure 2 shows an example of learning curves for the case of EfficientNetB0 as a baseline network for deep CNN feature extractor. The training loss functions showed a monotonic decreasing pattern with respect to epoch. The validation loss functions showed a decreasing pattern at earlier epochs up to 10, while it showed an increasing pattern at epochs later than the 10th epoch. The increasing pattern reflects overfitting as expected, and we chose an appropriate epoch number based on the dip of the validation loss functions. This pattern was observed in both the LSTM and GRU networks. The bottom sub-figure illustrates the accuracy curves with respect to epoch. The training accuracy curves kept increasing in all three models, while the validation accuracy curves stayed at an accuracy value with little fluctuation. The cascaded CNN-RNN models (green and red) had relatively higher accuracy than the CNN-alone model (purple). These trends were observed in other base networks of MobileNet, ResNet50V2, and NASNetMobile.

**Figure 3:**
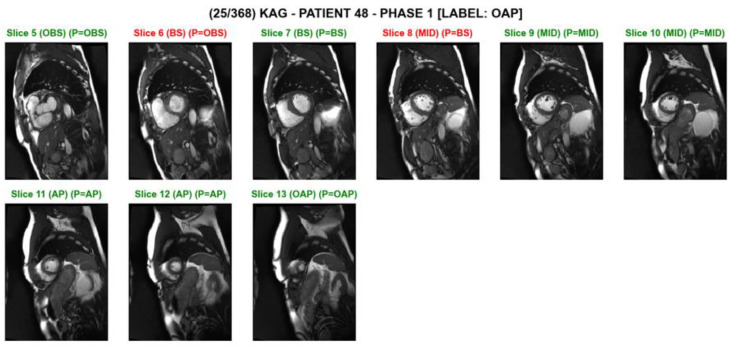
A screenshot of slice level predictions on a series of short axis slices on an individual test subject. The prediction was made by the ‘MobileNet_2layerLSTM’ model. The slice index, the ground truth, and the predicted category are shown above each image. The green text indicates correct classification, while the red text indicates incorrect classification. For example, ‘(BS) (P = OBS)’ indicates that the ground truth is ‘basal’, and the model’s prediction is ‘out-of-basal’.

Table 5 compares prediction results on testing data in a variety of CNN and CNN-RNN models. For a given baseline network, all four CNN-RNN models outperformed the CNN model in all the categories. Among the four baseline models considered, MobileNet produced the highest F1-scores in four out of the five classes. NASNetMobile was relatively poor in predictions. The MobileNet 2-LSTM model resulted in the highest scores in four out of the seven evaluation metrics. The MobileNet Bi-LSTM model resulted in the highest scores in two out of the seven evaluation metrics. Our Python implementation was effective in labeling each slice image as well as in checking the prediction results along with ground truth, as shown in Figure 3. In particular, the model comparisons were possible at an individual subject level. Bidirectional recursive temporal encoders generally outperformed their two-layer unidirectional counterparts, despite parity in model capacity. This indicates that the task of cardiac slice classification can benefit from bidirectional temporal modeling. The performance gap is highlighted in the oap category, e.g., the difference in F1-score between 2-GRU and bi-GRU for NASNetMobile is highest in oap, with a delta of 0.068 F1-score. This can be attributed to the absence of sequential signal from the unidirectional encoder, as there are no image slices that precede the oap category. This further supports the need for sequential modeling in the cardiac cine short axis classification task.

**Figure 4:**
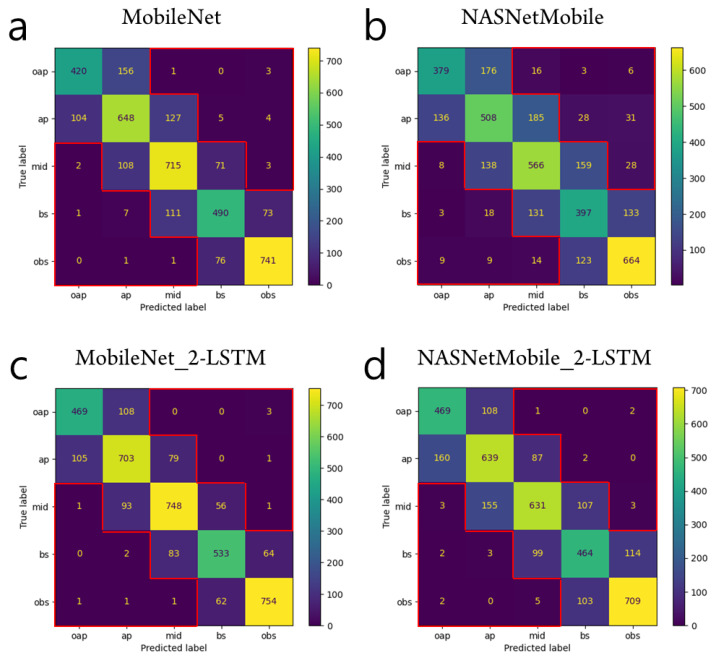
Test prediction results from the CNN-alone models (**a**,**b**) and from the CNN-RNN models (**c**,**d**). In either MobileNet or NASNetMobile, the CNN-RNN model has smaller sums of the elements out of the tridiagonal entries (SOTD) than the CNN-alone model. The elements out of the tridiagonal entries are indicated by the red contours in the confusion matrices. For example, SOTD is 11 for the case of MobileNet_2-LSTM in (**c**).

**Figure 5:**
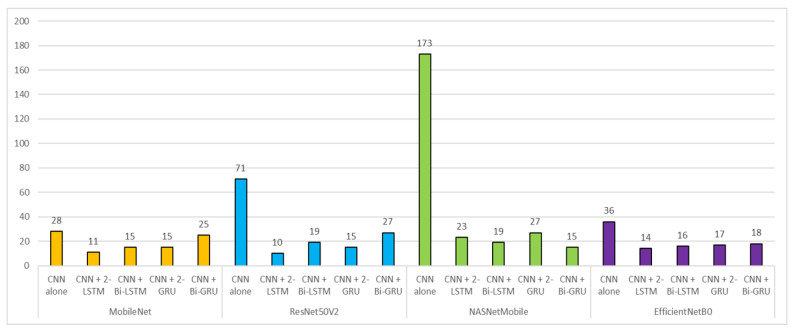
Comparison of SOTD values across deep learning models. SOTD was calculated to be the sum of the elements that are outside the tridiagonal entries. SOTD values were grouped by the type of the baseline deep CNN. The lower the SOTD value, the higher the prediction performance in general.

The confusion matrices in Figure 4 clearly show that the cascaded CNN-RNN models are superior to the CNN-alone model in predicting slice levels. SOTD quantification results showed the superiority of the cascaded CNN-RNN models in all the baseline networks. Regardless of the choice of baseline network, CNN-alone architecture resulted in larger SOTD than any of CNN-RNN models (see Figure 5). The lowest SOTD of 10 was observed in the ResNet50V2 CNN + two-layer LSTM model, and the highest SOTD of 173 was observed in the NASNetMobile CNN-alone model. This reduction in SOTD, i.e., errors with a class disparity of two or more, indicates that the cascaded models are better able to learn the similarity between adjacent slice categories. This may be attributed to the sequential modeling of RNN layers, which is able to leverage the adjacency information in the cardiac cine training data. The difference in SOTD between LSTM and GRU or between two-layer and bidirectional models was negligible in all the baseline architectures.

## 4. Discussion

The identification of cardiac short axis slice levels is essential in quantifying biomarkers such as ejection fraction, end-diastolic volume, and end-systolic volume and in precisely diagnosing myocardial disease based on the heart geometry in cardiac imaging. Automatizing this identification process improves efficiency in image analysis, and this study aimed to develop an automatic classification method based on a cascaded model of CNN and RNN. Validation on the test dataset resulted in the superior performance of the cascaded CNN-RNN model over the CNN-alone architecture regardless of the choice of a base deep CNN network model as a feature extractor.

A rigorous comparison of the proposed method against the previous method [7] was not straightforward because of the following two facts. First, the datasets used were different (Kaggle data vs. Cardiac Atlas data [29]). Second, the number of classes in the current study was five (i.e., oap, ap, mid, bs, and obs), whereas the number of classes in the previous study was three (i.e., oap, in, and obs). When merging the ‘ap’, ‘mid’, and ‘bs’ classes to the ‘in’ class and comparing the classification errors, we could compare the classification error rates between the previous method [7] and the proposed method. From the confusion matrix of the MobileNetV1 (alpha = 0.25) model in Figure 4b of the reference [7], the error rate was calculated as 14.7%. From the result of the MobileNet_2-LSTM model in Figure 4c, the error rate was calculated as 9.0% after merging the ‘ap’, ‘mid’, and ’bs’ classes to the ‘in’ class. Overall, the proposed cascaded CNN-RNN model was clearly advantageous over the CNN-alone model because it modeled spatial relationship in a stack of short axis slices.

We used the baseline deep CNN models as feature extractors. It would be worth investigating layer-wise fine-tuning to check for any improvements compared to the fixed feature extraction model [7]. In this study, we considered only four baseline models. When using the Keras library, we noted that other popular models such as VGG-Net [30] and DenseNet [31] had different image output configurations, and these models required more sophisticated handling of image preprocessing. Hence, we did not consider other deep CNN models with different image pre-processing configurations. Since our purpose was to confirm the benefit of the CNN-RNN architecture over the CNN-alone architecture in the short axis classification task, the use of the four popular networks would be sufficient for evaluating our comparison study. In addition, data augmentation could be an option for improving the prediction performance. It is noted that a custom design would be needed for implementing a data generator for the cascaded CNN-RNN models. The stack of short axis slice images should undergo the same transformation process when performing random rotation, translation, horizontal/vertical flip, or zoom-in/out during training. In addition, we considered only the Kaggle dataset in this study. Other publicly available datasets, such as the cardiac MRI Segmentation Challenge dataset, can be considered along with the Kaggle dataset, especially when one needs to perform more rigorous external validation with a different institution’s data [29].

Three-dimensional (3D)-CNN [32] is also a viable and popular approach to dealing with sequential image data. There are three main differences between 3D-CNN and CNN-RNN. Firstly, CNN-RNNs can leverage standard deep CNN architectures pre-trained on large-scale image datasets. This has been shown to be beneficial for medical applications, where training data are limited [7,33]. Secondly, RNNs are specifically designed for sequential modeling and are beneficial for long-term sequence modeling. The GRU and LSTM models used in our study employ gating mechanisms specifically aimed to preserve information in long-term sequences and aid training using long sequences by mitigating the vanishing gradient problem [34]. In contrast, 3D-CNNs are based on convolutions which have limited receptive fields, i.e., can only model the relationship between a few adjacent pixels or image slices covered by a kernel or sequence of kernels. To increase the receptive fields in CNNs, it is required to increase the number of layers, and even this has been shown to have a limited effect [35]. Finally, 3D-CNNs model the spatial and sequential characteristics of 3D samples in a joint manner, while the proposed CNN-RNN models these features in two separate steps. While 3D-CNNs can identify more fine-grained relationships that involve both spatial and sequential components in tandem, they require more computation and are harder to optimize compared to the 2D CNNs used in the CNN-RNN model. We believe that the task of cardiac slice classification does not require such fine-grained modeling. However, an in-depth study of this tradeoff is a promising direction for future work.

We investigated deep-learning-based identification of cardiac short axis levels on cardiac cine MRI data. This CNN-RNN prediction approach has other potential applications. Cardiac late gadolinium enhancement (LGE) has a similar stack of short axis slices, and this also requires precise identification of apical, mid, and basal slices for the visualization of a 17-segment model on a bull’s eye plot [36]. In addition, 3D images of cardiac perfusion [37] and T1 mapping [38] are acquired in a stack of short axis slices, and our cascaded CNN-RNN models would be a good fit for 3D perfusion and T1 mapping studies. It may be interesting to use pre-trained CNN-RNN models trained on cardiac cine data and evaluate them on unseen LGE, 3D perfusion, and 3D T1 mapping data to check for the effectiveness of transfer learning.

## 5. Conclusions

We developed and evaluated cascaded CNN-RNN models that take a series of short axis slices as input and predict a series of slice levels as output. When compared with the CNN-alone models, the cascaded CNN-RNN models resulted in higher F1-scores, AUC, and accuracy. A cascade of pre-trained MobileNet as the feature extractor and a two-layer LSTM network produced the highest scores in four out of seven evaluation metrics. This study was targeted on cardiac cine MRI, but the proposed method can be extended to other applications with similar imaging orientations, such as cardiac LGE, 3D myocardial perfusion, and 3D T1 mapping.

## Figures and Tables

**Table 1:** The numbers of subjects in training, validation, and testing groups.

	Training	Validation	Testing	Total
Number of subjects	576	214	184	974
Percentage (%)	59.1	22.0	18.9	100

**Table 2:** The numbers of samples in training, validation, and testing groups.

Model Type		Training	Validation	Testing	Total
CNN *	Number of samples	12,070	4594	3868	20,532
	Percentage (%)	58.8	22.4	18.8	100
CNN-RNN **	Number of samples	1152	428	368	1948
	Percentage (%)	59.1	22.0	18.9	100

* In the CNN model, the number of samples is defined as the total number of individual images. ** In the CNN-RNN model, the number of samples is defined as the total number of image series, where each set consists of a stack of short axis slices.

**Table 3:** The number of images for each class label in training and validation datasets.

		Class Label					Total
		oap	ap	mid	bs	obs	
Training	Number of images	1878	2784	2800	2132	2476	12,070
	Percentage (%)	15.5	23.1	23.2	17.7	20.5	100
Validation	Number of images	710	1086	1114	751	933	4594
	Percentage (%)	15.5	23.6	24.2	16.4	20.3	100

**Table 4:** The comparison of base deep CNN models.

CNN Base Network	Number of Model Parameters	ImageNet Top-1 Accuracy	Number of Features after GAP *	Batch Size for CNN	Batch Size for CNN-RNN
EfficientNetB0	5.3 M	77.1%	1280	32	2
MobileNet	4.2 M	70.6%	1024	32	2
NASNetMobile	5.3 M	74.4%	1056	32	2
ResNet50V2	25.6 M	76.0%	2048	16	2

* Global average pooling.

**Table 5:** Prediction performance of a variety of deep learning models. The bold indicates the highest value among the models.

	CNN Base Network	RNN Type	F1-Score					AUC *	Accuracy
			oap	ap	mid	bs	obs		
CNN	MobileNet	-	0.759	0.717	0.771	0.740	0.902	0.957	0.779
	ResNet50V2	-	0.748	0.668	0.726	0.688	0.868	0.944	0.740
	NASNetMobile	-	0.680	0.585	0.625	0.570	0.790	0.904	0.650
	EfficientNetB0	-	0.761	0.696	0.729	0.716	0.889	0.946	0.757
CNN-RNN	MobileNet	2-LSTM	0.811	0.783	**0.827**	**0.800**	0.918	**0.972**	**0.829**
		Bi-LSTM	0.825	0.779	0.812	0.793	**0.922**	**0.972**	0.827
		2-GRU	0.808	0.769	0.814	0.785	0.909	0.970	0.817
		Bi-GRU	0.819	0.784	0.801	0.784	0.907	0.970	0.819
	ResNet50V2	2-LSTM	0.759	0.763	0.804	0.759	0.904	0.966	0.801
		Bi-LSTM	0.821	0.781	0.782	0.769	0.908	0.968	0.812
		2-GRU	0.781	0.772	0.788	0.721	0.882	0.963	0.791
		Bi-GRU	0.816	0.746	0.755	0.758	0.909	0.962	0.796
	NASNetMobile	2-LSTM	0.771	0.713	0.733	0.683	0.861	0.952	0.753
		Bi-LSTM	0.809	0.713	0.772	0.711	0.874	0.960	0.777
		2-GRU	0.738	0.721	0.740	0.667	0.853	0.947	0.746
		Bi-GRU	0.806	0.747	0.770	0.712	0.869	0.958	0.780
	EfficientNetB0	2-LSTM	0.805	0.772	0.800	0.777	0.901	0.967	0.811
		Bi-LSTM	**0.827**	0.772	0.800	0.764	0.904	0.969	0.814
		2-GRU	0.811	0.763	0.793	0.764	0.909	0.965	0.808
		Bi-GRU	0.822	**0.785**	0.801	0.767	0.910	0.969	0.817

* Weighted AUC score. AUC: area under the curve.

## Data Availability

The data presented in this study are available on request from the corresponding author.
